# Outcomes of brace treatment for Adolescent Idiopathic Scoliosis - factors affecting the results of the treatment

**DOI:** 10.1186/1748-7161-8-S2-O51

**Published:** 2013-09-18

**Authors:** Toru Maruyama, Yusuke Nakao, Yosuke Kobayashi, Makoto Miura

**Affiliations:** 1 Saitama Medical Center, Saitama Medical University, Saitama, Japan

## Background

Controversies still exist regarding the effectiveness of brace treatment for adolescent idiopathic scoliosis (AIS). 

## Purpose

The goal of this study was to analyze the outcomes of brace treatment for AIS and to clarify the factors affecting the results of the treatment. 

## Methods

According to the Scoliosis Research Society (SRS) AIS brace studies standardization criteria, the following conditions were used to determine which patients to include in this study: aged 10 years or older, Risser 0-II, primary curve magnitude 25°-40° and no prior treatment when the brace was subscribed. At skeletal maturity, the percentage of patients whose curve progressed more than 6°, whose curve exceeded 45° or who underwent surgery was investigated. These results were compared with natural history or other brace studies. Factors affecting the results were analyzed. 

## Results

A total of 29 patients, 24 females and 5 males, met the SRS inclusion criteria. Average age at the beginning of the treatment was 12.0 years (range 10 to 15 years). Risser sign was 0 in 10, I in 6 and II in 13 patients. Curve pattern was thoracic (T) in 11, thoracolumbar or lumbar (TL) in 11 and double (D) in 7 patients. Average Cobb angle before treatment was 31.3°. Initial correction rate by the brace was 51.7% on average (35.2% for T, 75.5% for TL and 43.4% for D curve). Most patients wore their brace part-time, at home or at night. All the patients were followed until they reached skeletal maturity, with an average follow-up period of 29 months. The average Cobb angle at follow-up was 32.2°. Of 29 patients, six (21%) progressed more than 6° and three (10%) exceeded 45°. Only one patient (3%) underwent surgical treatment during the study period. These results were more favorable than the reported natural history or most of the other brace studies. Factors predicting the better results of brace treatment were Risser sign of I or II at the beginning of the treatment and better initial correction rate with the brace. 

## Conclusions and discussion

Of the curves with AIS, 79% were stabilized by the treatment. These results illustrate that brace treatment was effective for the treatment of AIS. 
